# The comprehensive checklist of myxomycetes of Ukraine, based on extended occurrence and reference datasets

**DOI:** 10.3897/BDJ.12.e120891

**Published:** 2024-04-11

**Authors:** Iryna Yatsiuk, Yuliia Leshchenko, Vitalii Viunnyk, Dmytro V. Leontyev

**Affiliations:** 1 University of Tartu, Tartu, Estonia University of Tartu Tartu Estonia; 2 Department of Ecology, Charles University, Prague, Czech Republic Department of Ecology, Charles University Prague Czech Republic; 3 Slobozhanskyi National Nature Park, Krasnokutsk, Ukraine Slobozhanskyi National Nature Park Krasnokutsk Ukraine; 4 Department of Botany, H.S. Skovoroda Kharkiv National Pedagogical University, Kharkiv, Ukraine Department of Botany, H.S. Skovoroda Kharkiv National Pedagogical University Kharkiv Ukraine

**Keywords:** historical literature, references, slime moulds

## Abstract

**Background:**

A significant body of valuable data about the myxomycetes of Ukraine lies in a “grey zone”. This encompasses undigitised historical books and articles published in languages such as Polish, French or German, as well as proceedings from local conferences, articles featured in local scientific journals and annual reports submitted to public authorities by employees of protected areas, published in Ukrainian or Russian. Yet, due to their exclusive existence in print and often the Cyrillic alphabet, these publications remain neither findable nor accessible to a wider audience.

**New information:**

The datasets presented here aim to summarise over 150 years of myxomycetes research in Ukraine. The majority of the data has been extracted from published literature sources spanning the years 1842 to 2023, with a minor supplement from unpublished herbarium specimens. The datasets include 5036 georeferenced occurrences, 339 taxa and 91 literature sources. Seventy-one of the used literature sources, mostly published before 2010, were uploaded to Zenodo and are available in open access.

## Introduction

Myxomycetes (Myxogastrea) are macroscopic terrestrial eukaryotes, capable of forming giant multinucleate plasmodia and fruiting bodies with a relatively complicated structure. Due to their conspicuous fruiting bodies, first documented observations of these organisms date back as far as the 17^th^ century (Leontyev et al. 2019).

First observations of myxomycetes in the territory of Ukraine appear in the first half of the 19^th^ century. In the monography named “Description of wild and domesticated plants in Lithuania, Volhynia, Podolia and Ukraine”, Polish Professor Józef Jundziłł reported 12 species of Myxomycetes (Jundziłł 1830). However, due to the absence of exact locality data in this publication, we cannot allocate these findings from historical regions to modern countries, which could be Ukraine, Poland, Belarus or Lithuania. The first report containing more precise geographic data was offered by a French mycologist Joseph-Henri Léveillé, known as the discoverer of a fungal basidium. He worked with herbarium specimens, collected in southern Crimea, on the coastline between Sevastopol and Nikita and identified two myxomycete species, namely *Lycogalaepidendrum* and *Stemonitisaxifera* (*Léveillé 1842*).

In 1845, the first mycological monograph written by a Ukrainian researcher was published. Professor Vasyl Chernyaev from the University of Kharkiv described 11 species and five genera of gasteroid fungi. One of them, *Xyloidiondelavignei* Czrern., is a forgotten synonym of *Lycogalaflavofuscum* (Leontyev 2023).

The first report on myxomycetes of the western part of Ukraine was published by Józef Krupa, who collected 75 species around Lviv and Stryi (Krupa 1886, Krupa 1887, Krupa 1889). During the same period, 20 species of myxomycetes were recorded in Chernihiv Province (Borszczow 1869) and eight species were found around Kyiv (Valtz and Rishavi 1871).

The publication of influential monographs by Józef Rostafinsky (Rostafinski 1875) and Arthur and Gulielma Lister (Lister 1894, Lister and Lister 1911) boosted studies of myxomycetes in Ukraine. In the period 1900−1914, slime moulds became a common part of local biodiversity research. At this time, R. Gutwinski (Gutwiński 1901) and B. Namyslowski (Namysłowski 1909) reported lists of fungi and myxomycetes from the historical region of Galicia. Although most of the records from these lists were spotted within the territory of modern Poland, several of occurrences can be georeferenced to western Ukraine. In the same time period, A. A. Jachevsky (Jachevsky 1907) reported myxomycetes from the central part of Ukraine and L. A. Benike (Benike 1914) in the east.

After the end of World War I and the Ukrainian Revolution, myxomycete research was resumed. M.O. Zelle, using her own materials, collections of V.I. Kazanovskyi and available publications, provided a preliminary checklist of the myxomycetes of the eastern and central parts of Ukraine, which included 63 species (Zelle 1925). A comprehensive review on the topic was published by M.M. Pidoplychko (Pidoplychko 1932). Using his own collections, herbaria of G.F. Borisevich and V.T. Panasenko, as well as thoroughly analysing literature sources, he published the annotated checklist of 67 species. During the same period, J. Jarocki reported 69 species from Chornohora in the Eastern Carpathians (Jarocki 1931). A. Namyslowska critically studied the herbarium of Prof. E. Lubich-Niezabitowsky collected near Stryi and found 61 myxomycete species (Namysłowska 1938). In the 1930s, renowned Polish myxomycetologist, H. Krzemieniewska, conducted surveys in western Ukrainian regions. Her research yielded the discovery of 83 species in the Carpathians and 46 species around Lviv (Krzemieniewska 1934, Krzemieniewska 1937).

Following the conclusion of World War II, research on myxomycetes in Ukraine came to a halt. For the period 1945−1991, only a few publications are known on this topic, including the species list of Kaniv Nature Reserve (Lavitska 1949) and “Handbook of the Fungi of Ukraine”, where 121 species of myxomycetes were listed, based on literature data, but with a first documented record of the *Licaethaliumolivaceum* (Zerova and Morochkovskyi 1967). In the 1980s, Y.K. Novozhilov conducted in southern Crimea a first investigation of myxomycetes, based on the moist chamber technique. Amongst reported 26 corticolous species, fourteen appeared to be new for Ukraine (Novozhilov 1986, Novozhilov 1988).

The resurgence of myxomycete research in Ukraine was instigated by Prof. I.O. Dudka. In 1994, she, together with her student T.I. Krivomaz, investigated the herbarium of H. Krzemieniewska deposited in Lviv National University and re-examined the data regarding the distribution of myxomycetes in the Ukrainian Carpathians (Dudka and Kryvomaz 1996). Working on the territory of the Carpathian Biosphere Reserve, together with B. Ing (UK), they recorded 45 species in this area. In 1995, I.O. Dudka and D. Minter (UK) published the preliminary checklist of the Fungi of Ukraine, which included data on 113 myxomycete species, kept in the National Herbarium of Ukraine, KW (Minter and Dudka 1996). In 2010, T.I. Kryvomaz published the first online database of the myxomycetes of Ukraine, where the distribution of species within the country was provided according to literature sources. Unfortunately, this database is not available on the web anymore.

Over the past two decades, the count of myxomycete species documented in Ukraine has doubled. The main efforts have been focused on the study of myxomycete biota in protected natural areas. Thorough surveys of corticolous (Kochergina and Markina 2021) and nivicolous (Leontyev et al. 2021) myxomycetes in preserved areas were published. Studies of the myxomycete-insect relationships have been conducted (Perkovskyi and Krivomaz 1994, Dudka and Romanenko 2006). Modern techniques, such as cultivation (Morozova 2010), biochemical analyses (Romanenko 2006), SEM (Leontyev and Moreno 2011, Leontyev and Fefelov 2012, Leontyev et al. 2015) and molecular barcoding (Leontyev et al. 2023, Yatsiuk et al. 2023) are now widely used by Ukrainian myxomycetologists.

In the period 2000−2010, scientific schools aimed at studying myxomycetes emerged in Kyiv and Kharkiv. Over ten Master theses, four PhD dissertations (Romanenko 2006, Leontyev 2007, Kryvomaz 2010, Kochergina 2021) and one DrSci dissertation (Leontyev 2016) were defended. Ukrainian collections were used for the description of 16 new species and two subspecies taxa; the holotypes of eight new taxa were collected in Ukraine. Two words of Ukrainian origin, Skovoroda (personal name) and palyanytsia (round bread) were used to create species epithets for new taxa (Leontyev and Fefelov 2009, Leontyev and Moreno 2011, Leontyev and Fefelov 2012, Leontyev et al. 2015, Leontyev et al. 2023, Yatsiuk et al. 2023).

With the onset of Russian aggression against Ukraine, many research programmes have been frozen and researchers moved abroad. However, myxomycete studies, including field research, are still ongoing. In 2022–2023, a previously unknown species of *Lamproderma* was found in the forests of the war-affected Kharkiv Region (Viunnyk and Leontyev 2023).

While the modern papers about myxomycetes of Ukraine tend to be written in English and are, generally, accessible online, a significant body of earlier data remains in a “grey zone”. This encompasses undigitised historical books and articles published in languages such as Polish, French or German, as well as proceedings from local conferences, articles featured in local scientific journals and annual reports submitted to public authorities by employees of protected areas, published in Ukrainian or Russian. These publications often provide lists of species recorded in specific localities in Ukraine, such as nature reserves, national parks or "zakazniks" (small areas with a limited conservation regime), constituting a valuable source of biodiversity data. Yet, due to their exclusive existence in print and often in the Cyrillic alphabet, these publications remain neither findable nor accessible to a wider audience.

Hence, this paper aims to provide a comprehensive summary of over 150 years of myxomycetes research in Ukraine and to enhance the accessibility of data from previously "invisible" sources.

Here, we introduce three datasets: the Checklist dataset (core), the Occurrence dataset, the Reference dataset (extensions).

- The occurrence dataset includes records mined from the scientific literature that make up to 98% of it. Another 2% of the occurrences represent, unpublished until now, parts of a herbarium collection of H.S. Skovoroda Kharkiv National Pedagogical University (CWP, Kharkiv, Ukraine), collected during the student summer practical fieldwork in 2017-2018 years. The occurrences retrieved either from literature or herbarium specimens were taxonomically assessed and, when necessary, georeferenced and quality-checked using the procedures described in Methods.
- The reference dataset includes digitised literature sources arranged in DarwinCore format. The list of literature sources encompasses 91 unique items. The essential part of the literature was procured from the institutional or private libraries and scanned starting from the 2000s by Dmytro Leontyev. Currently, it is stored in a digital form. The literature includes sources originally published in Ukrainian (38), Russian (21), English (22), Polish (8), German (1) or French (1) languages.
- The checklist is the final list of species derived from the occurrence dataset after the taxonomic assessment. New species, reported in recent publications, for which underlying data have already been published in GBIF, were added to the checklist individually.

These datasets represent the state of the art as of the beginning of 2023. Their purpose is to serve as a foundational resource for conducting countrywide ecological studies, particularly vital for evaluating the potential impact of the ongoing war on biodiversity in the future. The main limitation of the described datasets, primarily based on data from the pre-molecular era, lies in the discrepancy between historical myxomycete species identifications and modern classifications, based on molecular techniques. Additionally, it is important to note that all the literature sources included here are based exclusively on the fruiting body occurrences and exclude other stages in the myxomycete life cycle, like amoebae or plasmodia.

## Project description

### Title

Deciphering Cyrillic: the checklist from invisible sources

### Personnel

Yuliia Leshchenko, Iryna Yatsiuk

### Study area description

Ukraine

### Design description

BioDATA grant for data mobilisation including digitisation, data quality assurance, data preparation and publication of collection specimens and other species data from Ukraine to GBIF. Dataset preparation was supported within the project "Deciphering Cyrillic: the checklist from invisible sources". More details on the grant programme here (https://www.nhm.uio.no/english/research/projects/biodata/activities/data-mobilization-call-ukraine.html).

### Funding

- BioDATA partners, NLBIF, GBIF Norway and the UiO Natural History Museum;
- Cepa-LT-2017/10049;
- University of Tartu;
- Estonian Research Council project PRG1170 (author Iryna Yatsiuk).

## Sampling methods

### Sampling description

- The literature sources selected for this dataset included only scientific literature, including monographs, peer-reviewed journal articles, conference abstracts, annual reports of protected areas, PhD and Master’s theses. Herbarium specimens, included here, have either been identified or verified by academic myxomycetologists.
- The literature sources originally lacking a DOI, were published on Zenodo repository (zenodo.org) and assigned one. In total, 71 sources were published.
- Information from literature was extracted manually into comma-separated spreadsheets containing columns named according to the Darwin Core standard. To avoid duplication, sources, supported by datasets already published in GBIF, were not entered into the occurrence dataset, but added later at the stage of data analysis and checklist generation.
- The checklist dataset was created automatically by extracting unique values from the scientificName column within the occurrence dataset, following the completion of the taxonomic assessment.
- The References dataset was generated through manual extraction of information from literature sources, with the data organised into a comma-separated spreadsheet with the columns in the Darwin Core standard.

### Quality control

- Spreadsheets were checked and cleaned with Openrefine v. 3.2 (Ham 2013). Taxa names were checked for misspelling by matching against the GBIF Species Matching tool (https://www.gbif.org/tools/species-lookup). The results of georeferencing were checked visually by plotting occurrences with QGIS software (QGIS Development Team 2020).
- The taxonomic assessment was based upon “An online nomenclatural information system of Eumycetozoa” (Lado 2023), except in cases where subspecies or forms were reported. In instances involving subspecies/forms, as these are not recognised as valid taxa in the aforementioned nomenclature database, taxonomic treatments were based on several monographs (Martin and Alexopoulos 1969, Nannenga-Bremekamp 1991, Poulain et al. 2011), as well as expert taxonomic opinions.

### Step description

**The occurrence dataset** was produced with the following steps:

1. Survey and digitisation of professional literature resources on myxomycetes occurrences in the territory of Ukraine in its borders as of 1991 (total 91 sources);
2. Preparation of Darwin Core-formatted template;
3. Data extraction from the literature sources into corresponding columns;
4. Data extraction from the herbarium labels into corresponding columns;
5. Taxonomic assessment of names;
6. When necessary, automatic georeferencing of occurrences. If the coordinates of the occurrence were missing in the literature or on herbarium labels, the occurrence was georeferenced, based on text description of the occurrence location. Georeferencing was done automatically using the Geocode by Awesome Table extension for Google sheets (https://workspace.google.com/marketplace/app/geocode_by_awesome_table/904124517349). Precision was determined according to the accuracy of the distance to the occurrence from the authors' description in the text. The accuracy of the given coordinates is determined as follows: one number in decimal place corresponds to the precision of 11.1 km, two numbers = 1.11 km, with each subsequent sign, the distance is reduced by a factor of 10. In the case of localities indicated by names that have been renamed or no longer exist, georeferencing was carried out by the method of digitising available maps using QGIS 3.16.3 (QGIS Development Team 2020), followed by the extraction of coordinates. WGS84 was used as a spatial reference system.
7. Data cleaning using OpenRefine;
8. Matching species names;
9. Plotting of occurrences on the map and visual checkup of coordinates.

**The Reference dataset** was prepared with the following steps:

1. Preparation of Darwin Core-formatted template;
2. Data extraction from the literature sources into corresponding columns;
3. Data cleaning using OpenRefine.

**The checklist dataset** was derived from the occurrence dataset with the following steps:

1. Preparation of Darwin Core-formatted template;
2. Extraction of unique values from a scientificName column of the occurrence dataset to a scientificName of the checklist dataset.
3. Exclusion of identifications higher than species level (e.g. *Arcyria* sp.)

## Geographic coverage

### Description

The entire territory of Ukraine within 1991 borders.

**Figure 1.**
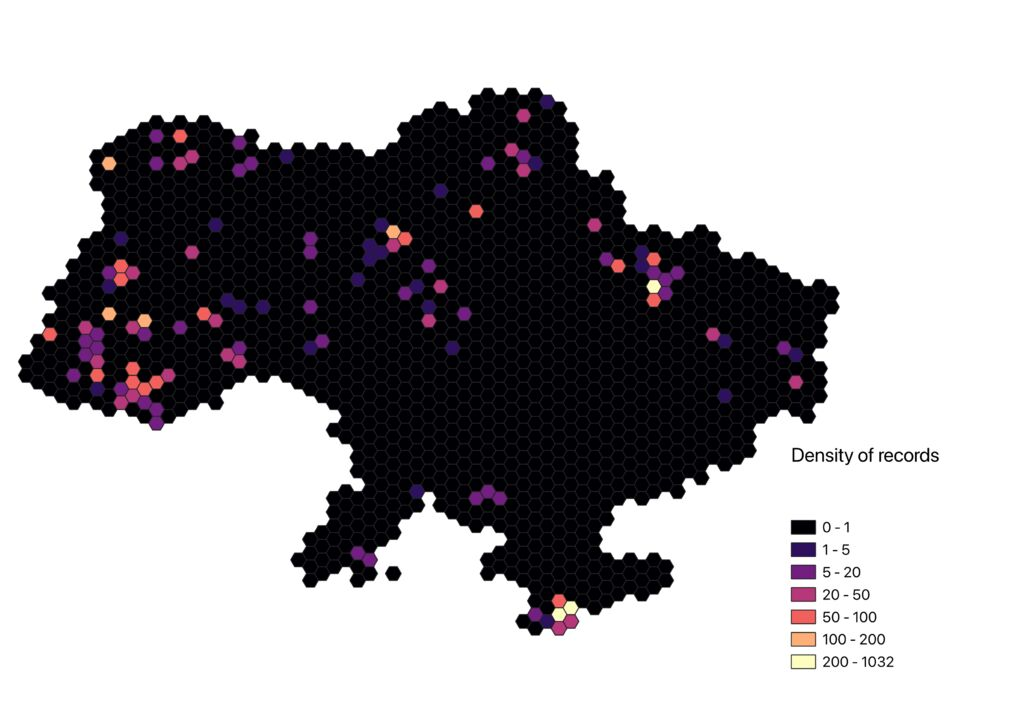
Distribution of occurrences of myxomycetes within the territory of Ukraine.

The distribution of occurrences in our dataset clearly mirrors the invested research effort (Fig. 1). The majority of records are concentrated in proximity to major cities and within protected areas situated in the forested regions of Ukraine, notably in the Carpathians, mountainous regions of Crimea and forest-steppe zone in eastern Ukraine. There is a noticeable data deficit in the southern regions of Ukraine, particularly in the steppe zone. Addressing this gap will necessitate dedicated research efforts, as well as collaboration with citizen scientists in the future.

### Coordinates

44.386389 and 52.379444 Latitude; 22.136944 and 40.227778 Longitude.

## Taxonomic coverage

### Description

**Figure 2.**
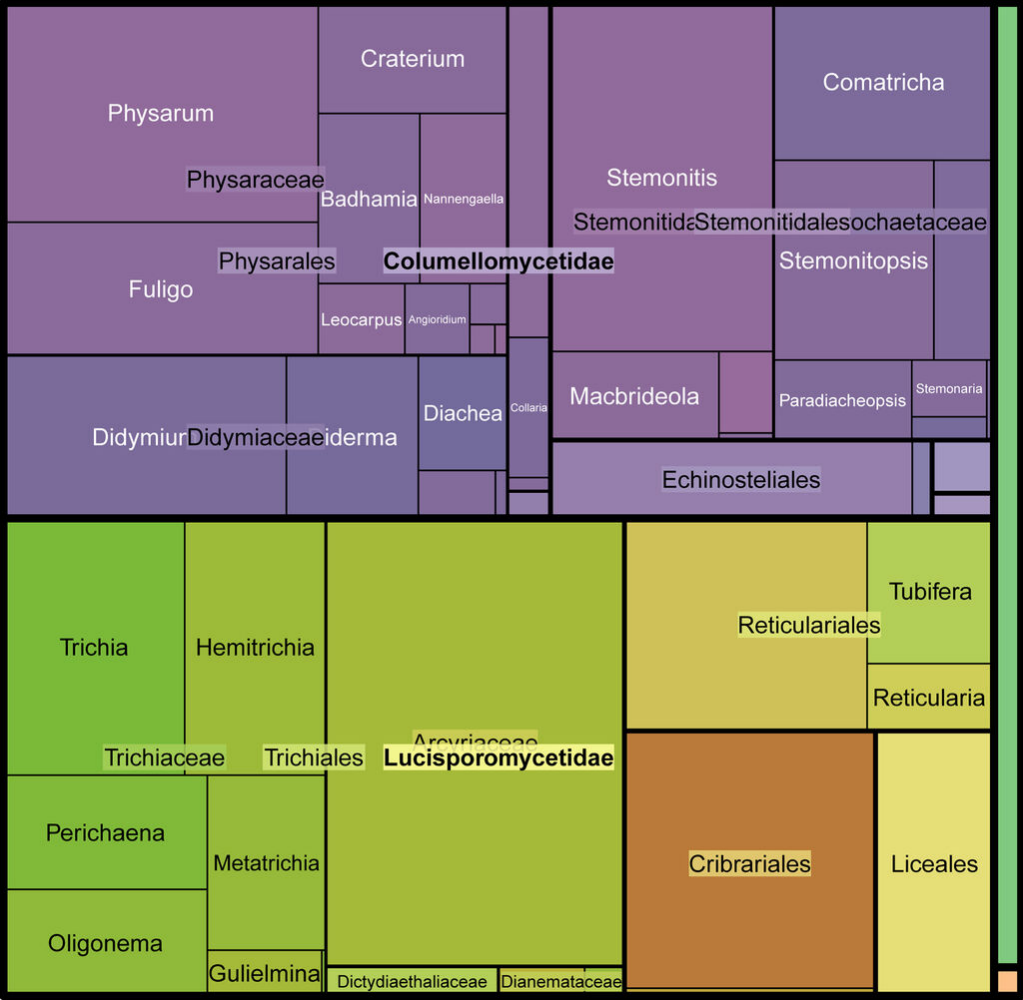
Taxonomic structure of occurrences of myxomycetes of Ukraine by the number of occurrences.

The checklist includes 339 taxa of slime moulds, amongst them, 331 are species and the rest are identified to the genus level. Nearly all records represent the class Myxomycetes (Eumycotozoa, Amoebozoa). One species belongs to the class Ceratiomyxomycetes (Eumycetozoa, Amoebozoa) and one represents the family Acrasidae (Heterolobosea, Excavata). While the latter one species, *Acrasisrosea*, is not even related to Eumycetozoa, it is a slime mould co-occuring with myxomycetes in moist chambers and is traditionally reported in the literature on myxomycetes of Ukraine. Subclasses Lucisporomycetidae (bright-spored) and Columellomycetidae (dark-spored) are represented by similar number of occurrences, 47% and 50% accordingly (Fig. 2). Amongst the genera of bright-spored myxomycetes, *Arcyria* (12.7%), *Cribraria* (6.7%) and *Lycogala* (5.7%) are recorded most often. For the dark-spored myxomycetes genera, *Physarum* (8.9%), *Stemonitis* (7.3%) and *Fuligo* (4.3%) are represented by the highest number of occurrences.

The estimated species richness, as determined by the bias-corrected Chao1 estimator, is 397 species. Consequently, the portion of known species accounts for 83% of the estimated overall diversity.

### Taxa included

**Table:** 

Rank	Scientific Name	
phylum	Eumycetozoa	
family	Acrasidae	

## Temporal coverage

### Notes

**Figure 3.**
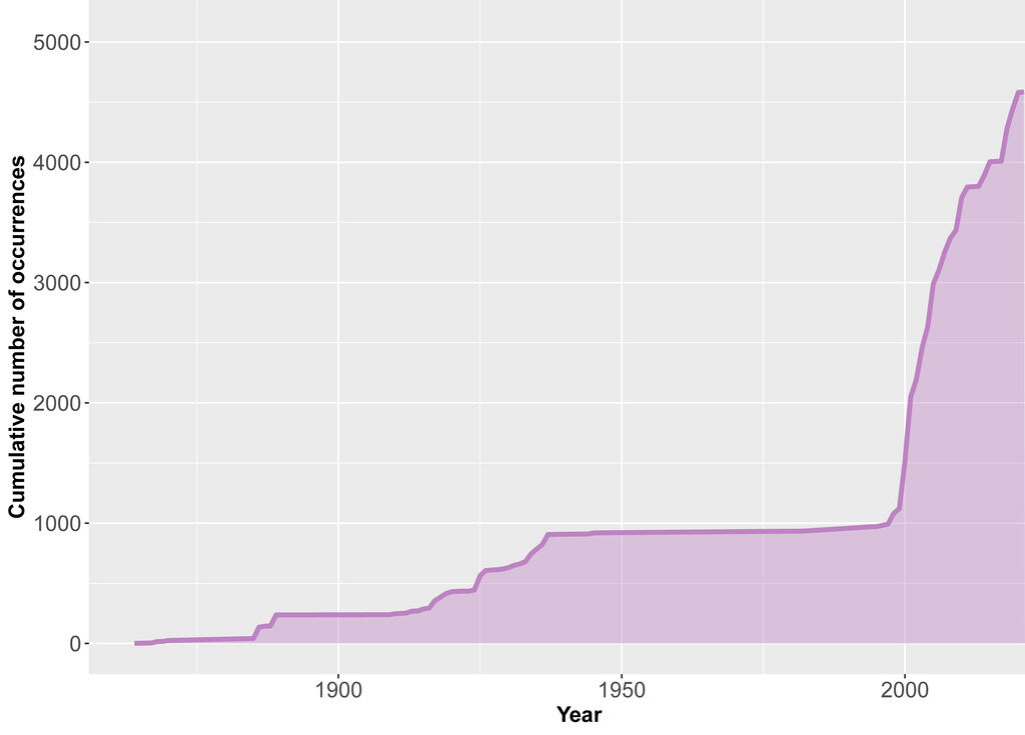
Temporal accumulation of records of myxomycetes in Ukraine.

Data range: 1830/1840s-2021 (Fig. 3)

**Figure 4.**
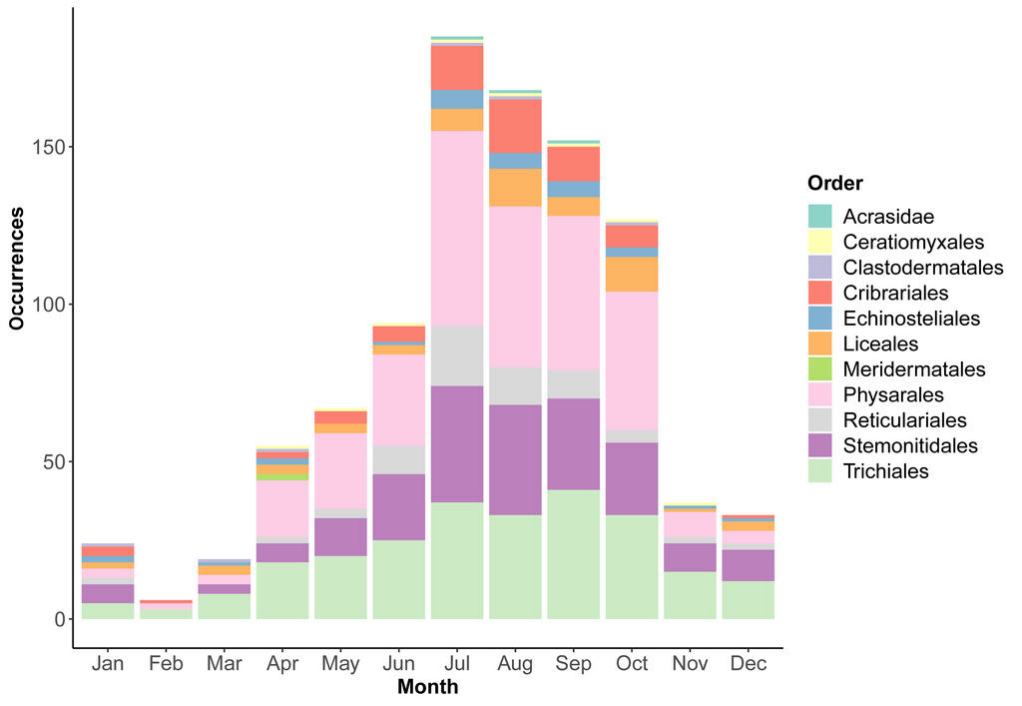
Distribution of occurrences of myxomycetes in Ukraine by month.

The majority of occurrences were registered in the late summer and early autumn months (Fig. 4). However, in April, there is a visible shift in taxonomic structure, namely increases in the share of the order Meridermatales. This reflects the specificity of nivicolous myxomycetes, the group of species that form fruiting bodies on the border of melting snow.

## Usage licence

### Usage licence

Open Data Commons Attribution License

## Data resources

### Data package title

The comprehensive checklist and extended occurrence and reference datasets of myxomycetes of Ukraine

### Resource link

https://doi.org/10.15468/7g9d74

### Alternative identifiers

https://ukraine.ipt.gbif.no/resource?r=myxomycetesukraine

### Number of data sets

3

### Data set 1.

#### Data set name

taxon.txt

#### Data format

Darwin Core

#### Download URL

https://www.gbif.org/dataset/7f90f977-1004-40f8-a471-7bbf517a006d

#### Description

The Taxon dataset includes a tabulation-delimited table with 11 fields in Darwin Core terms and 339 records.

**Data set 1.** 

Column label	Column description
taxonID	http://rs.tdwg.org/dwc/terms/taxonID; a unique identifier for the taxon, that serves as a foreign key amongst the three datasets presented here. The universal unique identifier (UUID) was used for this purpose.
taxonRank	http://rs.tdwg.org/dwc/terms/taxonRank; the lowest taxonomic rank of the occurrence in the acceptedNameUsage column.
scientificName	http://rs.tdwg.org/dwc/terms/scientificName; The full name of the currently accepted taxon, name after the taxonomical assessment performed as described in Methods.
kingdom	http://rs.tdwg.org/dwc/terms/phylum; The full scientific name of the kingdom according to the authoritative source.
phylum	http://rs.tdwg.org/dwc/terms/phylum; The full scientific name of the phylum according to the authoritative source.
class	http://rs.tdwg.org/dwc/terms/class; The full scientific name of the class according to the authoritative source.
order	http://rs.tdwg.org/dwc/terms/order; The full scientific name of the order according to the authoritative source.
family	http://rs.tdwg.org/dwc/terms/family; The full scientific name of the family according to the authoritative source.
genus	http://rs.tdwg.org/dwc/terms/genus; The full scientific name of the genus according to the authoritative source (https://eumycetozoa.com/).
specificEpithet	http://rs.tdwg.org/dwc/terms/specificEpithet; The species epithet of the dwc:scientificName.
scientificNameAuthorship	http://rs.tdwg.org/dwc/terms/scientificNameAuthorship; The authorship information for the dwc:scientificName formatted according to the conventions of the ICN.

### Data set 2.

#### Data set name

occurrence.txt

#### Data format

DarwinCore

#### Download URL

https://www.gbif.org/dataset/7f90f977-1004-40f8-a471-7bbf517a006d

#### Description

The Occurrence dataset includes a tabulation-delimited table with 24 fields in Darwin Core terms and 5036 records.

**Data set 2.** 

Column label	Column description
occurrenceID	http://rs.tdwg.org/dwc/terms/occurrenceID; the identifier for the occurrences, the universal unique identifier (UUID) was used for this purpose; the identifier for the occurrences, the universal unique identifier (UUID) was used for this purpose.
basisOfRecord	http://rs.tdwg.org/dwc/terms/basisOfRecord; MaterialCitation for the majority of occurrences derived from literature; PreservedSpecimen for herbarium records not cited in the literature.
catalogNumber	http://rs.tdwg.org/dwc/terms/catalogNumber; herbarium number of specimens.
eventDate	https://dwc.tdwg.org/terms/#dwc:eventDate; the full date of the observation as precisely as it could be extracted from the publication.
year	http://rs.tdwg.org/dwc/terms/year; the four-digit year in which the occurrence was recorded.
month	http://rs.tdwg.org/dwc/terms/month; month in which the occurrence was recorded.
day	http://rs.tdwg.org/dwc/terms/day; day of the month in which the occurrence was recorded.
verbatimEventDate	http://rs.tdwg.org/dwc/terms/verbatimEventDate; the original representation of the date/s in which the occurrence was recorded, mostly the dates referring to expeditions or surveys.
habitat	http://rs.tdwg.org/dwc/terms/habitat; description of macrohabitat such as vegetation type and microhabitat, such as a substrate type.
country	http://rs.tdwg.org/dwc/terms/country; one country (Ukraine).
countryCode	http://rs.tdwg.org/dwc/terms/countryCode; one value (UA).
stateProvince	http://rs.tdwg.org/dwc/terms/stateProvince; the highest-level administrative region of Ukraine (oblast’).
municipality	http://rs.tdwg.org/dwc/terms/municipality; the second-level administrative region of Ukraine, according to the official administrative division of Ukraine of 2020.
locality	http://rs.tdwg.org/dwc/terms/locality; more specific description of the locality then municipality, derived from the original.
minimumElevationInMetres	http://rs.tdwg.org/dwc/terms/minimumElevationInMeters;elevation a.s.l. if this information was stated in the original.
decimalLatitude	http://rs.tdwg.org/dwc/terms/decimalLatitude; geographic latitude in decimal degrees.
decimalLongitude	http://rs.tdwg.org/dwc/terms/decimalLongitude; geographic longitude in decimal degrees.
geodeticDatum	http://rs.tdwg.org/dwc/terms/geodeticDatum; The geodetic datum upon which the geographic coordinates given in dwc:decimalLatitude and dwc:decimalLongitude are based.
coordinateUncertaintyInMetres	http://rs.tdwg.org/dwc/terms/coordinateUncertaintyInMeters; the distance (in metres) from the given decimalLatitude and decimalLongitude describing the smallest circle containing the whole of the Location. Set as described in Methods.
georeferencedBy	https://dwc.tdwg.org/terms/georeferencedBy; name of the person who georeferenced the occurrence.
taxonID	http://rs.tdwg.org/dwc/terms/taxonID; a unique identifier for the taxon, that serves as a foreign key amongst the three datasets presented here. The universal unique identifier (UUID) was used for this purpose.
verbatimIdentification	http://rs.tdwg.org/dwc/terms/verbatimIdentification; A string representing the taxonomic identification as it appeared in the original record.
institutionCode	http://rs.tdwg.org/dwc/terms/institutionCode; The name (or acronym) in use by the institution having custody of the object(s) or information referred to in the record.
collectionCode	http://rs.tdwg.org/dwc/terms/collectionCode; The name, acronym, coden, or initialism identifying the collection from which the record was derived.
taxonRank	http://rs.tdwg.org/dwc/terms/taxonRank; the lowest taxonomic rank of the occurrence in the scientificName column

### Data set 3.

#### Data set name

reference.txt

#### Download URL

https://www.gbif.org/dataset/7f90f977-1004-40f8-a471-7bbf517a006d

#### Description

The Reference dataset includes a tabulation-delimited table with eight fields in Darwin Core terms and 2182 records.

**Data set 3.** 

Column label	Column description
identifier	http://purl.org/dc/terms/identifier; Digital Object Identifier was used for this purpose. If the publication originally was not assigned any DOI, it was issued by Zenodo after publishing it on zenodo.org.
bibliographicCitation	http://purl.org/dc/terms/bibliographicCitation; A text string referring to an un-parsed bibliographic citation.
title	http://purl.org/dc/terms/title; Title of the referenced item.
creator	http://purl.org/dc/terms/creator; The author or authors of the referenced item.
source	http://purl.org/dc/terms/source; the name of the journal, abstracts book or larger book (if any) where the item was published.
date	http://purl.org/dc/terms/date; the full date of the publication as precisely as it could be extracted from the publication.
language	http://purl.org/dc/terms/language; ISO 639-1 language code indicating the language of the publication.
id	equals to http://rs.tdwg.org/dwc/terms/taxonID (created automatically by the IPT as a replacement for taxonID); contains the unique identifier for the taxon that serves as a foreign key amongst the three datasets presented here. The universal unique identifier (UUID) was used for this purpose.

## Additional information

List of sources that were excluded from the occurrence dataset for various reasons are provided in Table 1.

## Figures and Tables

**Table 1.** List of sources that were excluded from the occurrence dataset.

**Source**	**Reason**
Jundziłł (1830)	Impossible to georeference records to the country level.
Namysłowski (1914)	Summary from other articles.
Glushchenko et al. (2002)	Textbook, no data about occurrences.
Dudka et al. (2009)	Summary from other articles.
Dudka et al. (2009)	Summary from other articles.
Yatsiuk et al. (2018)	Already published in GBIF, excluded from the Occurrences dataset to avoid duplication. However, data from this source are included in the taxon.txt, reference.txt and the analysis/vizualisations presented herein.
Yatsiuk and Leontyev (2020)	Already published in GBIF, excluded from the Occurrences dataset to avoid duplication. However, data from this source are included in the taxon.txt, reference.txt and the analysis/vizualisations presented herein.
Yatsiuk et al. (2023)	Already published in GBIF, excluded from the Occurrences dataset to avoid duplication. However, data from this source are included in the taxon.txt, reference.txt and the analysis/vizualisations presented herein.

## References

[B10893283] Benike L. A. (1914). The first information about the flora of mucous fungi in Kharkov and Kursk provinces. Protocols of the Society of Naturalists at the Imperial Kharkiv University.

[B10893364] Borszczow E. G. (1869). Ein Beitrag zur Pilzflora der Provinz Černigow. Bulletin de l'Académie impériale des sciences de St.-Pétersbourg.

[B10991592] Dudka II, Leontyev DV, Kryvomaz TA, Romanenko EA, Kochergina AV, university Perm state pedagogical (2009). Species and taxonomical diversity of the myxomycetes in forested protected ecosystems of Ukraine. The abstract book of the V International Conference "Studying of Fungi in Biocoenoses".

[B10893571] Dudka I. O., Kryvomaz T. I. (1996). New species of myxomycetes from the Ukrainian Carpathians. Ukrainian Botanical Journal.

[B10893417] Dudka I. O., Romanenko K. O. (2006). Co-existence and interaction between myxomycetes and other organisms in shared niches. Acta Mycologica.

[B10991555] Dudka I. O., Prydiuk M. P., Holubtsova Yu. I., Andrianova T. V., Karpenko K. K. (2009). Fungi and fungi-like protists of the National Nature Park "Desnyansko-Starohutskyi": monography.

[B10991547] Glushchenko V. I., Leontyev D. V., Akulov A. Yu (2002). Slime moulds.

[B10893426] Gutwiński R. (1901). Materiały do flory śluzowców (Myxomycetes) Galicji. Sprawozdanie Komisji Fizjograficznej.

[B10893435] Ham Kelli (2013). OpenRefine (version 2.5). http://openrefine.org. Free, open-source tool for cleaning and transforming data. Journal of the Medical Library Association: JMLA.

[B10893275] Jachevsky A. A. (1907). Mycological flora of European and Asian Russia. Volume 2. Slime moulds..

[B10893310] Jarocki J. (1931). Mycetozoa from the Czarnohora Mountains in the Polish Eastern Carpathians. Bulletin de l’Academie Polonaise des Sciences.

[B10893444] Jundziłł Stanisław Bonifacy (1830). Opisanie roślin w Litwie, na Wołyniu, Podolu i Ukrainie dziko rosnących iako i oswoionych.

[B10893588] Kochergina A. V. (2021). Corticolous myxomycetes (Myxogastrea) of the south-western part of Central Russian Upland: biodiversity and substrate ecology: PhD Dissertation: 091 – Biology.

[B10893452] Kochergina A. V., Markina (2021). Ecological assemblages of corticulous myxomycetes in forest communities of the North-East Ukraine. Biosystems Diversity.

[B10893461] Krupa Józef (1886). Zapiski mykologiczne przeważnie z okolic Lwowa i z Tatr. Kosmos, Ser. A, Biol..

[B10893479] Krupa Józef (1887). Zapiski mykologiczne z okolic Lwowa i Podtatrza. Sprawozdanie Komisji Fizjograficznej.

[B10893470] Krupa Józef (1889). Zapiski mykologiczne przeważnie z okolic Lwowa i Karpat stryjskich. Sprawozdanie Komisji Fizjograficznej.

[B10893596] Kryvomaz T. I. (2010). Taxonomic structure and aspects of ecology of myxomycetes in the forests of Ukraine: summary of the PhD Thesis in Biology: 03.00.21.

[B10893328] Krzemieniewska H. (1934). Śluzowce Karpat Wschodnich. Kosmos.

[B10893337] Krzemieniewska H. (1937). Śluzowce zebrane w starym ogrodzie botanicznym we Lwowie. Kosmos.

[B10893233] Lado Carlos (2023). An on line nomenclatural information system of Eumycetozoa.. http://www.nomen.eumycetozoa.com.

[B10893346] Lavitska Z. G. (1949). Materials for the flora of slime molds (Myxomycetes) of the Middle Dnieper region. Protocols of Kaniv biogeographic reserve.

[B10893400] Leontyev Dmitry V., Schnittler Martin, Stephenson Steven L. (2015). A critical revision of the *Tubiferaferruginosa* complex. Mycologia.

[B10893223] Leontyev Dmitry V., Schnittler Martin, Stephenson Steven L., Novozhilov Yuri K., Shchepin Oleg N. (2019). Towards a phylogenetic classification of the Myxomycetes. Phytotaxa.

[B10893497] Leontyev Dmitry V., Schnittler Martin, Kochergina Anastasia V. (2021). Nivicolous myxomycetes of the Carpathian National Nature Park: species and ribotypes. Nova Hedwigia.

[B10893355] Leontyev Dmytro (2023). *Xyloidiondelavignei* Czern., a forgotten synonym of *Lycogalaflavofuscum* (Ehrenb.) Rostaf.. Slime Molds.

[B10893488] Leontyev Dmytro, Ishchenko Yury, Schnittler Martin (2023). Fifteen new species from the myxomycete genus Lycogala. Mycologia.

[B10893215] Leontyev D. V. (2007). Myxomycetes of the National Nature Parl "Homilsha forests", summary of the PhD Thesis in Biology: 03.00.21.

[B10893382] Leontyev D. V., Fefelov K. A. (2009). Tubulifera
applanata, a new Myxomycete species from Eastern Europe and Northern Asia. Bulletin de Sociedad Micologica de Madrid.

[B10893373] Leontyev D. V., Moreno G. (2011). Reticularia dudkae. A new myxomycete species from oak forests of eastern Ukraine. Bulletin de Sociedad Micologica de Madrid.

[B10893391] Leontyev D. V., Fefelov K. A. (2012). Nomenclatural status and morphological notes on *Tubiferaapplanata* sp. nov. (Myxomycetes). Mycotaxon.

[B10893604] Leontyev D. V. (2016). Myxomycetes of the family Reticulariaceae: molecular phylogeny, morphology and systematics: summary of the Dr. Sci. Thesis in Biology.

[B10893506] Léveillé J. H., de Demidoff A. N. (1842). Voyage dans la Russie meridionale et la Crimee, par la Hongrie, la Valachie et la Moldavie..

[B10893519] Lister Arthur (1894). A monograph of the Mycetozoa, being a descriptive catalogue of the species in the herbarium of the British Museum. Illustrated with seventy-eight plates and fifty-one woodcuts.

[B10893257] Lister Arthur, Lister Gulielma (1911). A monograph of the Mycetozoa: a descriptive catalogue of the species in the Herbarium of the British Museum.

[B10893249] Martin G. W., Alexopoulos C. J. (1969). Slime Molds: The Myxomycetes..

[B10893527] Minter D. W., Dudka I. O. (1996). Fungi of Ukraine. A preliminary checklist.

[B10893612] Morozova I. I. (2010). Myxomycetes of the Nature Reserve "Medobory". Thesis for MA degree in Biology..

[B10893319] Namysłowska Aniela (1938). Śluzowce zebrane w okolicach Stryja przez profesora dra Edwarda Lubicz-Niezabitowskiego / Les myxomycètes recoltés dans les environs de Stryj (Sous-Carpates Orientales) par le Prof. Dr Edouard Lubicz-Niezabitowski. Sprawozdanie Komisji Fizjograficznej.

[B10893535] Namysłowski Bołesław (1909). Zapiski grzyboznawcze z Krakowa, Gorlic i Czarnej Hory. Sprawozdanie Komisji Fizjograficznej.

[B10991529] Namysłowski B. (1914). Śluzowce i grzyby Galicyi i Bukowiny. Pamiętnik Fizyograficzny.

[B10893207] Nannenga-Bremekamp N. E. (1991). A guide to temperate Myxomycetes..

[B10893620] Novozhilov Yu. K. (1986). Myxomycetes of the USSR. III. Genus Macbrideola H. C. Gilbert. Mycology and Phytopathology.

[B10899621] Novozhilov Yu. K. (1988). Epiphytic myxomycetes of some regions of the USSR. Analysis of distribution by types of substrates and habitats. Mycology and Phytopathology.

[B10893629] Perkovskyi E. E., Krivomaz T. I. (1994). Myxomycetes - food objects of four species of leiodids from the genera *Anisotoma* and *Agathidium* (Coleoptera, Leiodidae) in the Kaniv Reserve.. Bulletin of Zoology.

[B10893301] Pidoplychko M. M. (1932). Critical materials for the flora of myxomycetes of Ukraine. The Journal of the Biobotanical Cycle of the Academy of Sciences of the Ukrainian SSR.

[B10893199] Poulain M., Meyer M., Bozonnet J. (2011). Les myxomycètes.

[B10893409] Team QGIS Development (2020). QGIS Geographic Information System. Open Source Geospatial Foundation Project. http://qgis.osgeo.org. http://qgis.osgeo.org.

[B10893647] Romanenko K. O. (2006). Myxomycetes of the Crimean Nature reserve. PhD Thesis in Biology: 03.00.21.

[B10893241] Rostafinski Józef Tomasz (1875). Sluzowce (Mycetozoa).

[B10893562] Valtz Y. Y., Rishavi L. (1871). List of the collection of myxomycetes and fungi collected by A.S. Rogovich, Ya. Ya. Valtz and L. Rishavi. Notes of the Kyiv Society of Naturalists.

[B10976417] Viunnyk Vitalii, Leontyev DV. (2023). First records of bryophylous myxomycetes in the plane part of Ukraine..

[B10991538] Yatsiuk Iryna, Leontyev Dmitry, Shlakhter Mykhailo (2018). Myxomycetes of National Nature Park Slobozhanskiy (Ukraine): biodiversity and noteworthy species. Nordic Journal of Botany.

[B11033192] Yatsiuk Iryna, Leontyev Dmytro (2020). Two species of nivicolous myxomycetes that formed fruiting bodies during three spring seasons in the lowlands of the Eastern Ukraine. Phytotaxa.

[B10893265] Yatsiuk Iryna, Leontyev Dmytro, López-Villalba Ángela, Schnittler Martin, Kõljalg Urmas (2023). A new nivicolous species of *Lamproderma* (Myxomycetes) from lowland and mountainous regions of Europe. Nova Hedwigia.

[B10893292] Zelle M. O. (1925). Materials for the myxomycete flora of Ukraine. Bulletin of the Kyiv Botanical Garden.

[B10893580] Zerova MY, Morochkovskyi SF (1967). The identification guide for the fungi of Ukraine. Slime moulds (Myxophyta); Fungi (Mycophyta): Archimycetes, Phycomycetes..

